# A Pathway toward a New Era of Open-Cell Polyurethane Foams—Influence of Bio-Polyols Derived from Used Cooking Oil on Foams Properties

**DOI:** 10.3390/ma13225161

**Published:** 2020-11-16

**Authors:** Maria Kurańska, Elżbieta Malewska, Krzysztof Polaczek, Aleksander Prociak, Joanna Kubacka

**Affiliations:** Department of Chemistry and Technology of Polymers, Cracow University of Technology, Warszawska 24, 31-155 Krakow, Poland; elzbieta.malewska@pk.edu.pl (E.M.); krzysztof.polaczek@doktorant.pk.edu.pl (K.P.); aleksander.prociak@pk.edu.pl (A.P.); joannakubacka10@gmail.com (J.K.)

**Keywords:** green open-cell polyurethane foams, bio-polyols, modified used cooking oil, spray foams

## Abstract

In order to create greener polyurethane (PUR) foams, modified used cooking oils (UCO) were applied as starting resources for the synthesis of bio-polyols. The bio-polyols were produced using transesterification of UCO with diethylene glycol (UCO_DEG) and triethanolamine (UCO_TEA). Next, open-cell PUR foams were synthesized by replacing 20, 40, 60, 80 and 100% of the petrochemical polyol with the bio-polyol UCO_DEG or UCO_TEA. It was observed that an increasing bio-polyol content (up to 60%) led to an increase of the closed cell content. However, a further increase in the bio-polyol content up to 100% resulted in foam cell opening. The bio-foams obtained in the experiment had an apparent density of 13–18 kg/m^3^. The coefficient of thermal conductivity was determined at three different average temperatures: 10, 0 and −10 °C. The PUR bio-foams modified with bio-polyol UCO_TEA had lower values of thermal conductivity, regardless of the average temperature (35.99–39.57 mW/m·K) than the foams modified with bio-polyol UCO_DEG (36.95–43.78 mW/m·K). The compressive strength of most of the bio-foams was characterized by a higher value than the compressive strength of the reference material (without bio-polyol). Finally, it was observed that the bio-materials exhibited dimensional stability at 70 °C.

## 1. Introduction

Spray polyurethane (PUR) foams are most distinguished by a high content of closed cells (>90%). However, when an appropriate surfactant is used, cells can be opened in the foaming process and it is possible to obtain foams with a significant open-cell share or even fully open-cell materials. In recent years, an increase of interest in open-cell spray PUR foams has been observed [1]. The open-cell structure of a material offers high moisture vapor permeability, which in turn allows for applications of such foams in the attics of buildings. Open-cell PUR foams are also characterized by a lower apparent density than closed-cell ones. Conventional commercial open-cell PUR systems are derived from petrochemical raw materials. With people’s increasing awareness of the necessity to protect the environment, it has become important to explore renewable raw materials. In the chemical industry, a milestone in environmental protection was the introduction of the “green chemistry” idea in 1991 and one of its postulates is the implementation of renewable raw materials [2].

Several bio-renewable raw materials, such as natural oil and fillers, have drawn a lot of attention in the context of PUR production [3,4,5,6,7]. However, when it comes to application potential, vegetable oils are the most promising materials for the production of spraying PUR systems. Most vegetable oils, before being introduced into PUR systems, have to be modified in order to introduce hydroxyl groups. One of the methods of vegetable oil functionalization is epoxidation of carbon-carbon double bonds and subsequent oxirane ring-opening with proton donors [8]. The synthesis of bio-polyols using oxirane ring opening is often associated with oligomerization reactions leading to high viscosity, secondary hydroxyl groups (relatively low reactivity) and a low hydroxyl number of the final bio-polyols [9]. The synthesis method that offers bio-polyols with primary hydroxyl groups is transesterification of natural oil using different polyfunctional alcohols or amines [10,11].

Studies concerning closed-cell polyurethane foams modified with bio-polyols have shown that there are still challenges to produce these materials. When the share of polyol coming from vegetable oil reaches 100%, the foam properties are worse than those of the materials prepared using a mix of petroleum–based raw materials [9,12,13]. So far, the effect of the bio-polyol content on the characteristics of open-cell PUR foams having an apparent density of less than 20 kg/m^3^ has not been analyzed by other research groups. In our earlier work we analyzed the influence of bio-polyols originating from municipal waste with different hydroxyl values (140 and 159 mgKOH/g) and viscosities (3275 and 961 mPa·s) on the properties of open-cell PUR foams with apparent densities in the range of 12–17 kg/m^3^. The bio-polyols derived from municipal waste were made by epoxidation and ring-opening reactions with diethylene glycol. It was concluded that despite a clear difference between the properties of the synthesis components, the characteristics of the final open-cell polyurethane foams are alike [1].

It has been demonstrated that waste oil can be a valuable feedstock in the synthesis of polymeric materials, including polyurethanes [14,15,16]. Zhang and al. reported on the influence of liquefied products of peanut shell prepared in different conditions on the properties of open-cell PUR foams. The materials analyzed in their work had an apparent density of ca. 80 kg/m^3^ and were dedicated to be applied as floral foams [17].

In the literature, the effect of the chemical structure of bio-polyols obtained using transesterification of waste oil on the cell structure and properties of low-density (<20 kg/m^3^) open-cell PUR foams has not been described. Therefore, this work investigates the effect of the bio-polyol type and content in a PUR system on the properties of semi-rigid open-cell foams with a very low apparent density.

## 2. Materials and Methods

### 2.1. Foam Formulation

**Figure 1:**
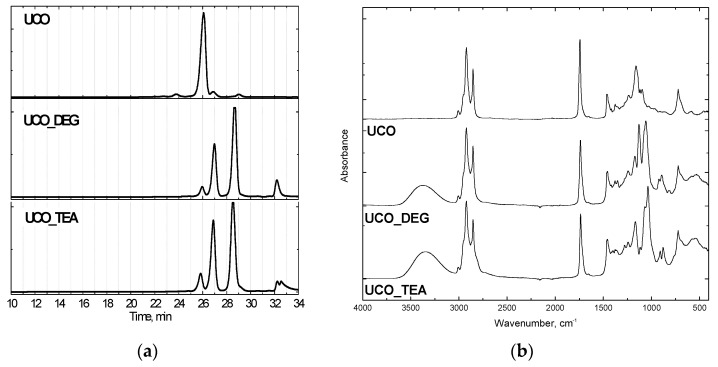
GPC chromatograms (**a**) and FTIR spectra (**b**) of bio-polyols.

Rigid PUR foams were made using polyol Rokopol 551 (oxypropylenated sorbitol, PCC Rokita, Brzeg Dolny, Poland), which was substituted to some extent by the bio-polyols based on waste oil (UCO_TEA or UCO_DEG). Characteristics of the bio-polyols, GPC chromatograms and FTIR spectra are shown in Table 1 and Figure 1.

The bio-originating polyols were synthesized from used cooking oil following the transesterification method with diethanolamine (UCO_TEA) and diethylene glycol (UCO_DEG) at Cracow University of Technology. The reagents were introduced with a molar ratio of 1:3 (waste oil: transesterification agent). The reaction was carried out at a temperature of 175 °C and the catalyst content (zinc acetate) was 0.3 wt.%. The reaction product consists of monoglycerides, diglycerides and triglycerides (Figure 1a). Used cooking oil was amassed from restaurants. The GPC (, Warsaw, Poland) -based chromatograms showed characteristic peaks of triglycerides (26 min), diglycerides (27 min) and monoglycerides (29 min). Additionally, the FTIR (Perkin Elmer, Waltham, MA, USA) analysis attested to the chemical structure of the bio-polyols. The broad band at 3340–3390 cm^−1^ corresponded to free hydroxyl groups in the polyols (Figure 1b). PCC Rokita SA provided petrochemical polyether polyol, and the hydroxyl value of this material was 440 mgKOH/g.Polymeric methylene diphenyldiisocyanate (Ongronat 2100), with a free isocyanate groups content of 31.5 wt%, was offered by Borschodchem. Evonik supplied catalysts for the foaming (dimethylaminoethoxyethanol) and gelling (dibutyltin dilaurate) reactions and also a surfactant. The reaction of water with isocyanate groups generated carbon dioxide, which served as a chemical blowing agent. The content and isocyanate index of each component are shown in Table 2.

### 2.2. Preparation of Samples

PUR foams containing bio-polyol UCO_TEA or UCO_DEG in various amounts were produced following a one-step method from components A and B. The mixture of polyols, amine catalyst, surfactant and water was mechanically stirred for 15 s until their full homogenization was reached. Next, a certain amount of isocyanate (component B) was introduced into the polyol premix (component A) so that the molar ratio of the NCO to OH groups was 1:1. Then, the system was mechanically mixed for 3 s and poured into an open mold. The resultant foams were conditioned for 24 h at room temperature. The sample names were coded as OPU_TEA_X or OPU_DEG_X, where X means the amount of the bio-polyol in the polyol premix (per hundred polyol).

### 2.3. Characterization of Bio-Polyols, Foaming Process and Foam Properties

The contents of hydroxyl and acid groups were found as recommended by the PN-93/C-89052/03 and PN-EN ISO 660:2009 standards, respectively. A GPC analysis was carried out to determine the number and weight average molecular weight as well as dispersity. A Knauer chromatograph equipped with a PLgel MIXED-E column and also a refractometric detector were applied in the analysis of oligomers. A rotational rheometer HAAKE MARS III (Thermo Scientific, Waltham, MA, USA) was used at a temperature of 25 °C to find viscosity. In this case, the plate-plate arrangement in the control rate mode was selected, with 20 mm-diameter plates and rotation speeds of 100 cycles per minute. The PN-81/C04959 standard was followed to find the content of water in the bio-polyols.

The foaming process was analyzed using FOAMAT (Format Messtechnik GmbH, Karlsruhe, Germany)—a foam qualification system. This system can determine the most important parameters: dielectric polarization, temperature, pressure and foam growth velocities. Temperature was measured by means of thin thermocouples. A foam pressure measurement device was applied to observe changes of pressure. Dielectric polarization was investigated using a curing monitor device (CMD, Format Messtechnik GmbH, Karlsruhe, Germany). The device gives an insight into the electrochemical processes occurring in foam formation. Dielectric polarization reflects the conversion degree of functional groups during a PUR formation. A scanning electron microscope (HITACHI TM3000, Tokyo, Japan) and optical microscope (PZO, Warsaw, Poland) revealed the morphology of cells. Image analysis was done thanks to a dedicated piece of software Aphelion^TM^ (Version 3.1). The content of closed cells in the foams was determined in accordance with the ISO 4590 standard, whereas the apparent density was in line with PN-EN ISO 845. The thermal conductivity coefficients were found using a Fox 200 laser comp heat flow instrument (New Castle, DE, USA). The temperatures of the cold plate were −10, 0 and 10 °C while the temperatures of the warm plate were chosen to be 10, 20 and 30 °C to achieve average temperatures of 0, 10 and 20 °C, respectively, and a constant temperature difference of 20 °C between the plates. The compressive strength was found in line with PN-EN 826. The compressive force was applied at a speed of 2 mm/s, axially in a normal direction to a square surface. The compressive stress was calculated at 10% deformation. The mechanical properties of the foams were investigated parallel (pa) and perpendicular (pe) to the foam rise direction. Measurements were carried out on five samples. The dimensional stability was calculated using the formula recommended in the PN-92/C-89083 standard. The dimensional stability of the PUR foams was tested 24 h after sample conditioning at +70 °C and −25 °C.

## 3. Results and Discussion

Foaming is the most important stage during PUR foam preparation. The reaction mixture during this process increases its volume several dozen times and the polymer matrix in the foamed bio-materials described in this paper constitutes ca. 1–1.5 vol.%.

**Figure 2:**
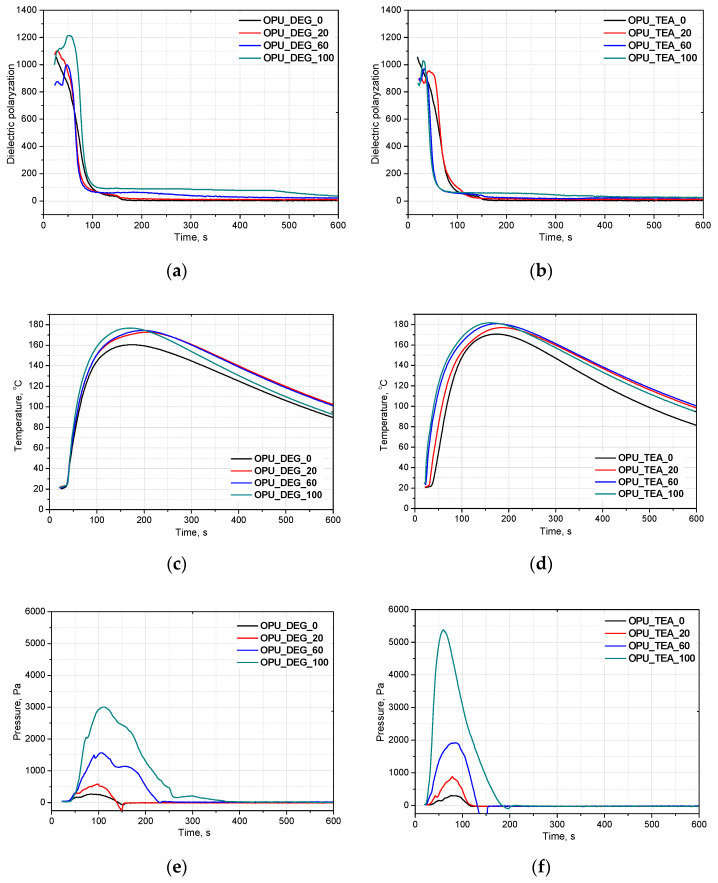
Bio-polyols UCO_DEG and UCO_TEA with different contents and their influence on on dielectric polarization (**a**,**b**), temperature (**c**,**d**) and pressure (**e**,**f**) of reaction mixtures.

Appropriate timing of the gelling and foaming reactions determines the formation of a regular cell structure that affects mechanical and thermal insulating properties of foams. Composing new systems based on renewable raw materials requires a detailed analysis of the influence of bio-components on foaming. FOAMAT allows one to follow the reaction and how it is affected by bio-polyols. The foaming parameters are measured in a cylindrical container placed on a foam pressure measurement device containing a built-in dielectric polarization sensor. Dielectric polarization is a key value in measuring chain formation and cross-linking of PUR foams. A dielectric polarization sensor is made of two comb shaped electrodes forming a plane capacitor. This sensor is integrated with a pressure measurement device located in the base plate on which the polyurethane system is poured. The expanding foam provides close contact with the sensor, which ensures direct penetration of the electric fringe field. Changes in the dielectric polarization are directly connected with the capacity of the sensor. The dielectric polarization is generated by dipole moments of molecules with bipolar structures (OH groups of polyols, NCO groups of isocyanates). The chain formation and cross-linking reaction ultimately suppress dipole mobility. The dielectric polarization tracks the formation of intermediates like amines and polyurea before they are bonded to the PUR matrix, as well as the final foam curing visible as a low, stable signal. Figure 2 shows the effect of a different content of bio-polyol UCO_DEG (**a**) and UCO_TEA (**b**) on the dielectric polarization (**a**,**b**), temperature (**c**,**d**) and pressure (**e**,**f**) in the foaming process of the PUR systems in our experiment.

Based on the decrease of the dielectric polarization of the reactions, it was concluded that independently of the bio-polyol UCO_DEG content, the reactivity of the PUR system is comparable. In the case of the PUR system modified with bio-polyol UCO_TEA, higher reactivity was observed for systems OPU_TEA_60 and OPU_TEA_100. In our previous work, we showed that PUR systems derived from bio-polyol with triethanolamine are characterized by higher reactivity due to the presence of nitrogen atoms. The PUR foams had a closed-cell structure and apparent density of about 37 kg/m^3^ [18]. It can be concluded that the reactivity of a PUR system is not affected by the type of foams (open- or closed cell) or their apparent density. In the system modified with bio-polyol UCO_DEG, the reactivity was similar to that of the reference system and this may be an effect of primary hydroxyl groups in the bio-polyol structure. In our earlier work, basd on a measurement of dielectric polarization, we showed that bio-polyols containing secondary hydroxyl groups caused a decrease of the reactivity of most PUR systems [1,18,19]. However, an opposite effect was described in one article [20]. The application of bio-polyol with secondary hydroxyl groups made the foaming and gelation reactions faster than in the reference material. However, this unexpected effect could be related to higher viscosity of the bio-polyol used [20].

**Figure 3:**
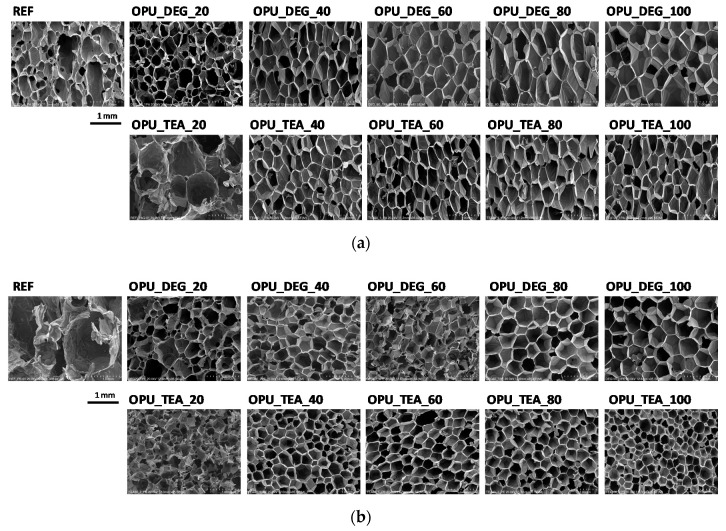
SEM images of foams cell structure in cross-sections: (**a**) parallel and (**b**) perpendicular with respect to direction of foam rise.

**Figure 4:**
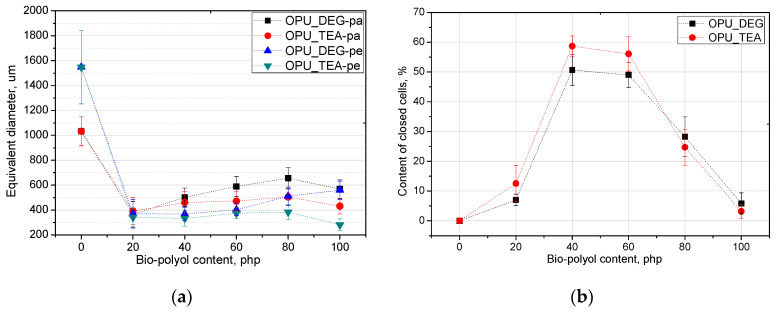
Equivalent diameter (**a**) and closed cell content (**b**) of bio-foams altered with bio-polyols in different amounts.

The PUR formation reactions are highly exothermic. The rate of temperature rise determines the reactivity of PUR systems [21]. The temperature in the reaction mixture core is measured be placing a thermocouple in the lower part of a mold (in practice one third of the final height of the foam). All the modified systems were characterized by a higher maximum temperature than the reference PUR system during the foaming process and it could also be correlated with higher pressure during their expansion. The trapped blowing agents are heated which causes the gas pressure to increase, creating stresses inside the foam. The stress of the expanding foam loads the bottom of the cylinder where the applied force is measured by a pressure sensor. The course of the foaming process has a strong influence on the foams cellular structure, especially in the materials with apparent densities lower than 20 kg/m^3^. In Figure 3, SEM images of the PUR cellular structure are shown. Figure 4 presents the influence of the bio-polyol type and content on the average cell diameter (**a**) and closed cell content (**b**).

The content of closed cells and their size have a noticeable impact on the thermal insulation and mechanical properties of the foams. The replacement of petrochemical polyols, regardless of their content, led to a decrease of the cell size of the bio-foams as compared to the reference material. It was observed that the foams produced with bio-polyol UCO_TEA had a lower value of the cell diameter. This effect can be related with a more branched structure of bio-polyol UCO_TEA and a higher reactivity of the PUR system modified with this bio-polyol. Bio-polyols based on natural oil can also act as plasticizers of a PUR matrix due to the presence of dangling chains in their structure. Such an effect is described in the literature for closed-cell PUR foams [22,23]. However, in this paper it was shown that such an effect was also observed for open-cell PUR foams.

**Figure 5:**
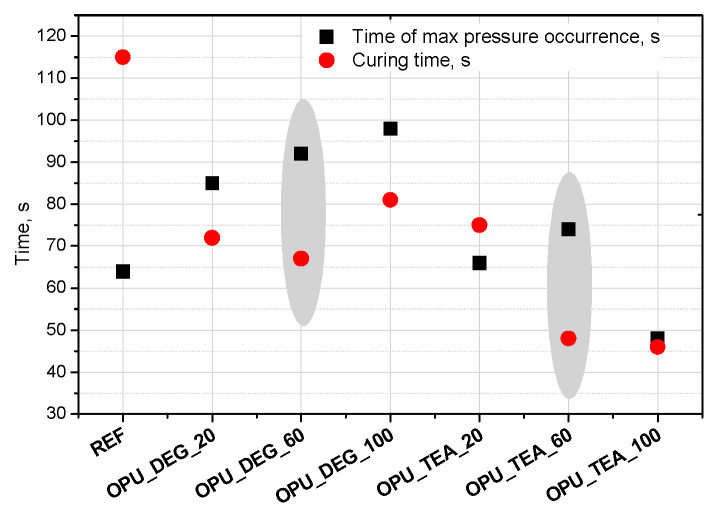
Time of max pressure occurrence and curing time of polyurethane (PUR) systems.

Interesting results were obtained as far as the closed cell content of the bio-foams is concerned. A tendency to create closed cells was noticed for the systems modified with the bio-polyols in an amount of 20, 40 and 60%. When the bio-polyol content reached 80% in the PUR system, the cells again exhibited a tendency to open. The OPU_TEA_100 material was characterized by an open-cell structure (closed cell content <5%). This effect may be connected to the cross-linking time, phase separation which induced weakness in the polymer membranes between pores and the time of peak pressure (Figure 5).

Curing time is evaluated from the dielectric polarization curve. It was found that in the case of the materials with a 60% content of the bio-polyol in the premix (regardless of its type), the greatest difference between these times was observed (Figure 5). The time of curing was significantly shorter compared to the time of maximum pressure, which made it difficult to open the cells in the cross-linked foam. However, in the case of the reference foam this effect was not observed. This may be due to the irregular cellular structure (large cells), which caused inaccurate adherence to the measuring device.

The content of closed cells, alongside other important properties, significantly affects the thermal conductivity coefficient of PUR foams. In general, the heat transport mechanism in porous materials is complex. The total thermal conductivity of foams is constituted by contributions from gas cells, the polymer matrix and the radiative heat transfer within the foam, which are not additive. In the case of an open-cell foam, the gas present in cells is air. Moreover, the convection of gases in porous PUR materials with an open-cell structure is high, in contrast to closed-cell foams [24]. The thermal conductivity of open-cell PUR foams is in the range 0.037–0.039 W/(m·K) [1]. In Table 3, the thermal conductivities of the reference foams and the foams built on cooking oil-based polyols are shown.

**Figure 6:**
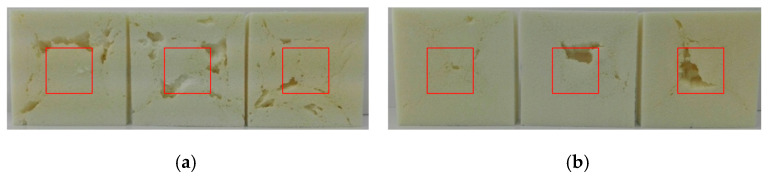
Photographs of PUR samples OPU_DEG_20 (**a**) and OPU_DEG_40 (**b**).

The thermal conductivity depends strongly on the average temperature of the measurement. The higher the temperature, the higher the conductivity coefficient. Generally, producers of PUR foams provide the value of the thermal conductivity coefficient at an average temperature of 10 °C. All the foams modified with the UCO_TEA bio-polyol have thermal conductivity values comparable to those of commercial foams [3]. This is associated with the cellular structure of the foams based on bio-polyol UCO_TEA, which is characterized by a low equivalent cell diameter. In this work, it was confirmed that the reduction of the cell diameter reduced the thermal conductivity of open-cell foams. Until now, such a relationship has been described in the literature for closed-cell foams [25]. Piszczyk et al. concluded that a change in the pore diameter from 0.25 to 0.6 mm increases the value of thermal conductivity coefficient by almost 50% [24]. It was also observed that material OPU_DEG_40 had the highest coefficient of thermal conductivity. This effect is a result of a disturbance in the foam structure probably due to the addition of this bio-polyol. A similar disturbance was also observed in the case of the material modified with 20% of the OPU_DEG bio-polyol. Such effects could be caused by insufficient miscibility of the polyols used in the polyol premix. The more favorable thermal conductivity coefficient for the OPU_DEG_20 foam compared to the OPU_DEG_40 foam, despite the disturbances in the foam structure, resulted from the methodology of measuring the heat conductivity coefficient. The measurement was performed on the central surface of the material (as marked with a red square) and resulted from the specificity of the device used (Figure 6). In the case of the UCO_DEG_20 foam, despite the defects, the thermal conductivity coefficient had a favorable value due to the regular structure in the middle of the material. However, in UCO_DEG_40, the defect was within the measurement area in two out of the three foams, hence the much higher value of the standard deviation.

According to the literature, the compressive strength of closed-cell PUR foams is strictly correlated with their apparent density. Such a relationship is described in the literature for foams characterized by an apparent density > 35 kg/m^3^ [18,26,27]. However, the literature results indicate that the cellular structure and closed cell content of PUR foams also have a significant influence on their mechanical strength. Hejna et al. concluded that incorporation of bio-polyol produced by polymerization of crude glycerol and further condensation with castor oil led to an increase of the foam compressive strength, despite a decrease of the apparent density. It is caused by a reduction of the average cell size from 372 to 275 um [27].

**Figure 7:**
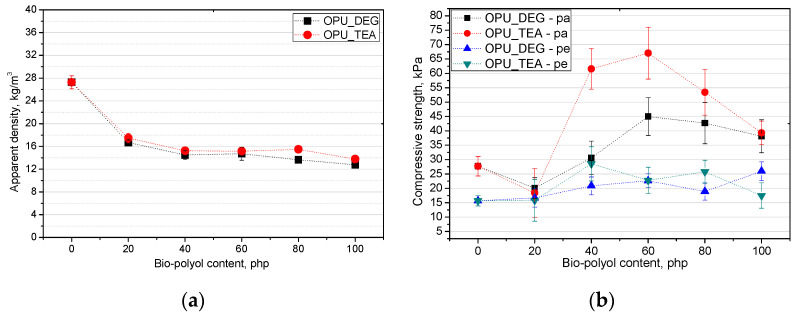
Influence of bio-polyols and their content on apparent density (**a**) and compressive strength (**b**) of modified foams.

In PUR foams the value of the compressive strength in a direction perpendicular to the foam rise direction is lower than in a parallel direction. This difference comes from the cell anisotropy and it is characteristic for closed and open-cell foams. The elongation of cells in the compression direction improves the mechanical properties, but causes a deterioration perpendicularly. In Figure 7, the influence is shown of the bio-polyol content on the apparent density (**a**) and compressive strength (**b**).

The compressive strength of commercial open-cell PUR foams is about 10 kPa. The low compressive strength results from the foam apparent density which is in the range of 7–14 kg/m^3^ [28]. The cell structure has a key influence on the mechanical properties. The size of the cells as well as their type (open or closed) is important. Figure 7b shows the change in compressive strength for the foams modified with the bio-polyols. It is shown that the tendency of the changes in the compressive strength was comparable with the changes in the case of the results of the closed cell content. The more closed cells there were, the higher the compressive strength became. The compressive strength of the porous materials containing the bio-polyols was higher than that of the reference material for most of the modified foams despite their lower apparent density.

**Figure 8:**
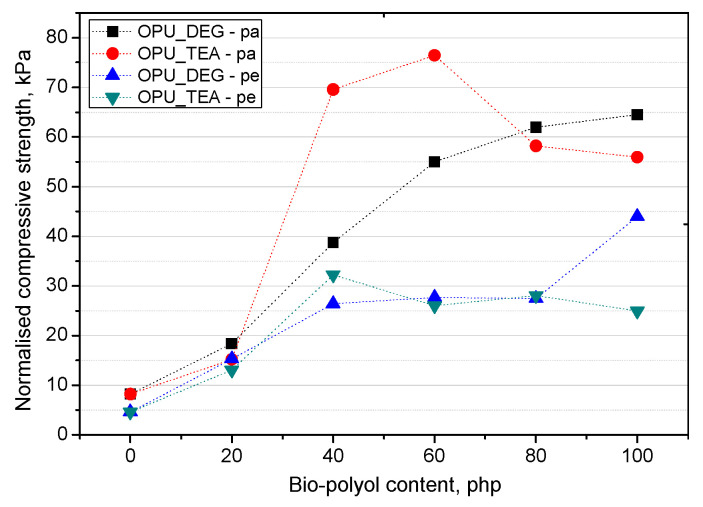
Normalized compression strength of PUR foams.

The mechanical properties were normalized to an average PUR foam apparent density of 16 kg/m^3^ (*σ_norm_*) according to the equation of Hawkins [29] in order to be able to compare their values. The equation for normalized compressive strength calculation is presented below:

$$\sigma_{norm}=\sigma_{exp}{\left(\frac{\sigma_{norm}}{\sigma_{sample}}\right)}^{2.1}$$

where *σ_exp_* is the experimentally determined compressive strength and *σ_sample_* is the PUR sample apparent density. The normalized compressive strength was calculated in directions parallel and perpendicular to the foaming rise direction (Figure 8).

On the basis of the normalized values of the compressive strength, it can be stated that when the petrochemical polyol was replaced with either type of bio-polyol, a beneficial effect was observed. An increase of the compressive strength was observed especially for the samples modified with 40–60% of the OPU_TEA bio-polyol. However, if the mechanical properties were measured in a more critical direction (perpendicular to the foam rise direction) the most favorable properties were obtained for the materials modified with 100% of the UCO_DEG bio-polyols. The mechanical properties perpendicular to the foam growth direction are very important due to their close correlation with the foam’s dimensional stability.

Despite the low values of the apparent density and compressive strength of the foams, the results obtained for the dimensional stability at elevated (70 °C) and low (−25 °C) temperatures were satisfactory (Table 4).

The size changes were lower than 1.5% regardless of the measurement direction. For the open-cell foams, the difference between the internal cell gas pressure within the foam and the external atmospheric pressure was zero and the dimensional stability problem is avoided. That was why, open-cell polyurethane foams can have a very low apparent density without shrinkage problems. Such a property makes them suitable for application as thermal insulation materials.

## 4. Conclusions

The objective of this work was to analyze the effect of bio-polyols made from waste cooking oil with different chemical structures on the properties of PUR foams. The foams were prepared by replacement of 20, 40, 60, 80 and 100% of a petrochemical polyol with the bio-polyols. It was found that the bio-polyol (regardless of its type) had a strong effect on the foam structure. An increase of the bio-polyol content (up to 60%) led to an increase of the closed cell content, yet, on the other hand, a further increase in the bio-polyol content (up to 100%) resulted in cell opening in the foams. The PUR foams had apparent densities from 13 to 18 kg/m^3^, depending on the weight ratio of the used cooking oil-based polyol. The thermal conductivity of the PUR materials ranged between 35.99 and 43.78 mW/m·K proportionally to the concentration of the bio-polyol.

The PUR bio-foams altered with bio-polyol UCO_TEA exhibited lower values of thermal conductivity. The modification with the bio-polyols had no clear effect on the dimensional stability of the PUR foams. The satisfactory results for the PUR foams with a high content of the bio-polyols show that waste cooking oil may serve as a valuable raw material in the synthesis of bio-polyols and can be successfully applied in the preparation of open-cell PUR foams.

## Figures and Tables

**Table 1:** Characteristics of the bio-polyols and petrochemical polyol.

Properties	UCO_TEA	UCO_DEG	Rokopol 551
Hydroxyl value, mgKOH/g	348	277	420
Acid value, mgKOH/g	2.31	1.35	0.10
Water content, % mas.	0.05	0.13	0.10
Average molecular weight, g/mol	522	492	~600
Viscosity, mPa·s	182	56	3000–5000
Functionality	~2.2	~1.9	~4.5

**Table 2:** Formulations of foams.

Raw Materials, g	REF	OPU_DEG_20 OPU_TEA_20	OPU_DEG_40 OPU_TEA_40	OPU_DEG_60 OPU_TEA_60	OPU_DEG_80 OPU_TEA_80	OPU_DEG_100 OPU_TEA_100
Rokopol 551	100	80	60	40	20	0
Bio-polyol UCO_DEG or UCO_TEA	0	20	40	60	80	100
Polycat 218	4	4	4	4	4	4
Tegostab8870	4.5	4.5	4.5	4.5	4.5	4.5
Ortegol 500	0.5	0.5	0.5	0.5	0.5	0.5
Water	15	15	15	15	15	15
Ongronat 2100	332	323.4	314.8	306.4	297.8	289.3
		328.1	324.2	320.3	316.4	312.4

**Table 3:** Coefficients of thermal conductivity of foams.

Symbol	Coefficient of Thermal Conductivity Measured at Average Temperature, mW/m·K:					
	0 °C		10 °C		20 °C	
REF	41.35	±0.17	43.90	±0.16	45.83	±0.57
OPU_DEG_20	36.95	±0.16	38.95	±0.50	40.05	±0.12
OPU_DEG_40	42.38	±0.63	43.60	±4.97	44.28	±1.52
OPU_DEG_60	37.93	±0.62	39.76	±0.23	41.62	±1.40
OPU_DEG_80	38.47	±0.32	40.16	±0.70	42.62	±0.78
OPU_DEG_100	39.15	±0.44	41.34	±0.58	43.17	±0.44
OPU_TEA_20	35.99	±1.62	37.63	±1.90	39.98	±2.05
OPU_TEA_40	36.00	±0.46	37.96	±1.06	39.93	±0.74
OPU_TEA_60	37.25	±1.50	39.57	±0.80	41.68	±0.72
OPU_TEA_80	37.18	±0.24	39.02	±0.43	41.61	±0.31
OPU_TEA_100	37.15	±1.41	39.06	±1.31	40.44	±1.22

**Table 4:** Dimensional stability of foams measured at 70 °C and −25 °C.

Symbol	Dimensional Stability at 70 °C after 24 h/48 h, %			Dimensional Stability at −25 °C after 24 h/48 h, %		
	Length	Width	Thickness	Length	Width	Thickness
REF	−0.15/0.12	−0.46/0.50	−0.52/0.04	−0.04/−0.34	0.07/−0.15	0.07/−0.97
OPU_DEG_20	0.22/0.24	0.51/0.30	0.49/0.46	0. 40/−0.01	0.07/−0.29	0.05/−0.68
OPU_DEG_40	0.37/0.33	0.33/0.24	0.69/0.43	0.03/−0.18	−0.07/−0.16	−0.20/−0.66
OPU_DEG_60	0.39/0.28	0.38/0.35	−0.30/−0.17	0.15/−0.04	−0.08/−0.24	−0.09/−0.60
OPU_DEG_80	0.61/0.56	0.45/0.22	0.34/0.07	−0.07/−0.26	−0.13/−0.22	−0.76/−1.26
OPU_DEG_100	−0.10/−0.25	0.12/−0.09	0.39/0.29	−0.06/−0.01	−0.11/−0.22	−0.40/−0.15
OPU_TEA_20	−0.48/−0.76	0.30/0.19	0.49/−0.06	0.04/−0.02	0.26/0.10	0.79/0.86
OPU_TEA_40	0.14/−0.06	0.12/−0.15	0.60/0.01	0.09/0.01	0.11/0.00	0.37/0.04
OPU_TEA_60	0.89/0.97	0.30/0.16	0.90/0.86	0.12/−0.01	0.13/0.09	0.28/0.22
OPU_TEA_80	0.16/−0.06	0.12/−0.10	−0.01/−0.42	−0.11/−0.15	0.02/−0.10	−0.12/0.14
OPU_TEA _100	1.46/1.36	1.37/1.29	1.65/1.47	−0.19/−0.42	−0.15/−0.29	−0.41/−0.42
